# Filariasis of parti-colored bats: phylogenetic analysis, infection prevalence, and possible vector mite identification

**DOI:** 10.3389/fvets.2025.1546353

**Published:** 2025-03-05

**Authors:** Sarka Bednarikova, Ondrej Danek, Heliana Dundarova, Monika Nemcova, Vladimir Piacek, Katerina Zukalova, Jan Zukal, Jiri Pikula

**Affiliations:** ^1^Department of Ecology and Diseases of Zoo Animals, Game, Fish and Bees, University of Veterinary Sciences Brno, Brno, Czechia; ^2^Department of Veterinary Sciences, Faculty of Agrobiology, Food and Natural Resources/CINeZ, Czech University of Life Sciences, Prague, Czechia; ^3^Institute of Biodiversity and Ecosystem Research, Bulgarian Academy of Sciences, Sofia, Bulgaria; ^4^Institute of Vertebrate Biology, Czech Academy of Sciences, Brno, Czechia

**Keywords:** *Vespertilio murinus*, onchocercid filarial nematode, *Litomosa*, vector-borne parasites, *Steatonyssus spinosus* mite, *Wolbachia*

## Abstract

**Introduction:**

The epidemiology of filarial infections is a neglected area of bat research, with little information on filarial species diversity, life cycles, host ranges, infection prevalence and intensity, parasite pathogenicity, or competent vectors. Furthermore, molecular data for filarial worms are largely lacking.

**Methods:**

Here, we examined 27 cadavers of parti-colored bat (*Vespertilio murinus*) from Czech rescue centers for filarial infection using gross necropsy. We also used nested polymerase chain reactions targeting partial mitochondrial cytochrome c oxidase subunit I (*COI*) partial gene to detect and genotype filarial parasites within organs, and ectoparasites of *V. murinus* from Russian and Slovak summer bat colonies. Samples with mixed filarial infections were cloned to extract separate sequences. The *COI* gene sequences were then subjected to phylogenetic analysis and a phylogenetic tree constructed. Adult filarial worms were also screened for the bacterial symbiont Wolbachia, using a standard PCR targeting the partial *16S rRNA* gene.

**Results:**

Two filarial nematode species were identified in single and mixed *V. murinus* infections, *Litomosa* sp. and a species of Onchocercidae. Adult *Litomosa* sp. nematodes were only recorded during necropsy of the abdominal, thoracic, and gravid uterine cavities of four bats. Molecular screening of organs for filarial DNA revealed prevalences of 81.5, 51.9 and 48.1% in *Litomosa* sp., Onchocercid sp. and co-infected bats, respectively. Adult *Litomosa* sp. worms proved negative for *Wolbachia*. The macronyssid mite *Steatonyssus spinosus*, collected in western Siberia (Russia), tested positive for Onchocercid sp. and mixed microfilarial infection.

**Discussion:**

Our results revealed high prevalence, extensive geographic distribution and a potential vector of filarial infection in *V. murinus*. Our data represent an important contribution to the field of bat parasitology and indicate the need for a taxonomic revision of bat-infecting filarial nematodes based on both morphological and molecular methods.

## 1 Introduction

Filarial nematodes are thread-like vector-borne parasites of medical and veterinary importance (1). To be able to reproduce, adults of both sexes must occur within their definitive vertebrate hosts. Gravid females are ovoviviparous, meaning that they release larvae (microfilariae) that spread through tissues and/or enter lymphatic and blood circulation (2). Transmission between hosts occurs after the microfilariae are ingested by a competent arthropod vector, and develop into L3 larvae to become infective (3). Filarial worms are commonly long-lived and many species show low pathogenicity, causing non-life-threatening infections in animals. However, there are a few exceptions, such as the canine heartworm (*Dirofilaria immitis*), which causes cardiopulmonary infection (1). Furthermore, filarial infections can be difficult to detect at their predilection sites and may be overlooked in asymptomatic individuals (4).

While filariae are somewhat neglected as chiropteran parasites, the two best-known onchocercid nematodes of bats are of the genera *Litomosa* and *Litomosoides* (5, 6). Two species have been identified in the parti-colored bat (*Vespertilio murinus*), *Litomosa ottavianii* (7) and *Litomosa vaucheri* (8). While *L. ottavianii* was described morphologically based on a few dozen females and males collected from *V. murinus* and common bent-wing bats [*Miniopterus schreibersii* (7)]; *L. vaucheri* is known only as a single intact female and as an anterior and posterior fragment of a female, both specimens without microfilariae, from *V. murinus* (8). *Litomosa ottavianii* have also been reported from greater horseshoe bats (*Rhinolophus ferrumequinum*) in Serbia (9). Identified as *Litomosa* sp. using a molecular assay targeting the cytochrome c oxidase subunit 1, filarial adults have recently been detected in the peritoneal cavity of a male *V. murinus* (10). Interestingly, microfilariae were present in both the semen and the testes of this bat. However, argasid mite larvae parasitic on the bat proved negative for filarial DNA, meaning that its arthropod vector remains to be identified. As morphological characteristics suggested a novel filarial species, and a full description of this new species has yet to be made (10), it has not yet been possible to link morphological and molecular identification in this case.

Many onchocercid nematode species co-evolved with the intracellular bacterial endosymbiont *Wolbachia*, which plays an essential role in their biology and may be a target for anti-filarial drug treatment (11, 12). However, adult *Litomosa* worms from the peritoneal cavity of the parti-colored bat tested negative for *Wolbachia* (10).

To date, nothing is known about the epidemiology of filarial infections in *V. murinus* (10). Here, we utilized cadavers of *V. murinus* obtained from Czech wildlife rescue centers, along with macronyssid mites collected from *V. murinus* captured in a Russian summer bat colony, to examine filarial infection prevalence and distribution in the host body. Alongside necropsy, we used DNA-based tools to detect and genotype filarial parasites, their bacterial endosymbiont *Wolbachia*, and to identify their potential natural vector. Given the necessity of increasing DNA amplification sensitivity, we developed a novel nested polymerase chain reaction (nested-PCR) assay for detection of filarial infection in bats. We then predicted host sex-related differences in infection prevalence in *V. murinus* bats based on different roosting abundances of female and male colonies.

## 2 Materials and methods

### 2.1 Sampling of bat cadavers and ectoparasites

Between 2010 and 2015, a total of 27 *Vespertilio murinus* cadavers were obtained from wildlife rescue centers around the Czech Republic (synanthropic habitats of the cities Prague) (50°5′15″N, 14°25′17″E), Brno (49°11′43″N, 16°36′30″E), Melnik (50°21′2″N, 14°28′27″E), Mnisek pod Brdy (49°52′0″N, 14°15′43″E). The cadavers were dissected and individual organ samples (testes, heart, spleen, kidneys, liver) and any adult worms found in the body cavities were removed and stored in 70% ethanol for further analysis. The examination of the bat cadavers did not reveal any ectoparasites. In contrast, live bats from Russia and Slovakia were examined only for ectoparasites, without the possibility of obtaining dead bats or other samples.

Ectoparasites of *V. murinus* were obtained from seven bats sampled from summer roosting colonies in Russia (Lukashino, western Siberia (57°19’N, 64°59′E), natural habitat; city of Voronezh (51°40′18″N, 39°12′38″E), southern Russia, synanthropic habitat) in 2018 to 2019, and Slovakia (Cierny Balog (48°44′50″N, 19°39′22″E), natural habitat) in 2022, the ectoparasites being removed with forceps and fixed in 70% ethanol for further analysis. In all cases, the bats were released close to their roosting sites immediately after sampling.

All 35 ectoparasites collected from the bats were determined based on morphological characteristics (13–16), and were represented by mites *Steatonyssus spinosus* (*n* = 27) and *Steatonyssus* sp. (*n* = 2), flea *Ischnopsyllus obscurus* (*n* = 5), and tick *Carios vespertilionis* (*n* = 1). The ectoparasites were then grouped into nine pooled samples according to their species and bat origin, five representing *S. spinosus*, two *I. obscurus*, one *C. vespertilionis*, and one comprising *Steatonyssus* sp.

### 2.2 DNA isolation

DNA was extracted from adult filarial worms, bat tissue (testes, heart, spleen, kidneys, and liver) and ectoparasites using the NucleoSpin® Tissue Kit (Macherey-Nagel, Germany), according to the manufacturer’s instructions. An Implen NanoPhotometer (Implen, Germany) was used to evaluate the quantity and quality of isolated DNA by calculating the absorbance ratio at 260 nm and 280 nm. The DNA samples were then stored at −20°C until further use.

### 2.3 Molecular assays for detection of filariasis and Wolbachia screening

Two PCR sets were used in this study: a nested-PCR targeting the partial gene of mitochondrial cytochrome c oxidase subunit I (*COI*), used for the detection and identification of filarial nematodes, and a standard PCR targeting the partial *16S rRNA* gene, used for the detection of *Wolbachia* endosymbionts in the DNA obtained from adult worms (*n* = 5). Both rounds of nested-PCR targeting *COI* were prepared in a total volume of 20 μL, comprising 10 μL of Phusion Green Hot Start II High-Fidelity PCR Master Mix (Thermo Fisher Scientific, USA), 0.5 μM of each primer (see Table 1), 2 μL of template DNA, and 6 μL of PCR grade water. The PCR targeting the *16S rRNA* gene was performed at a total volume of 25 μL, comprising 12.5 μL of Super-Hot Master Mix 2x (Bioron GmbH, Germany), 0.4 μM of each primer, 9.5 μL of PCR water, and 1 μL of template DNA. For further details on the primers and PCR protocols, see Table 1.

**Table 1:** Primers and reaction conditions used in the present study.

Marker	Primer	Sequence	Product length [bp]	Annealing Temp. [°C]	References
*COI*	CF F3	5’-TTCTGTTTTDACTATRCATGG-3’	957	53	This study
	CF R5	5’-GCHACAACATAATAAGTATCATG-3’			
	COI int. F	5′-TGATTGGTGGTTTTGGTAA-3’	689	53	(56)
	COI int. R	5’-ATAAGTACGAGTATCAATATC-3’			
*16S rRNA*	16S 281F	5’-CTATAGCTGATCTGAGAGGAT-3’	~1,100	55	(57)
	16S 1372R	5’-YGCTTCGAGTGAAACCAATTC-3’			

All PCR reactions were performed using a MJ Mini™ Personal Thermal Cycler (Bio-Rad Laboratories, USA), with a negative (PCR grade water) and positive (DNA isolated from *Dirofilaria repens*) control included in each run. The obtained PCR products were visualized on a 1.5% agarose gel stained with Serva DNA Stain G (Serva, Germany) under UV light. All PCR products of appropriate size were purified using the NucleoSpin® Gel and PCR Clean-up Kit (Macherey-Nagel, Germany), and then commercially sequenced using Sanger sequencing (SEQme s.r.o., Czech Republic). The obtained sequences were then aligned with available sequences in the GenBank database^1^ using MegaBLAST, and edited using Geneious Prime software (Biomatters Ltd., New Zealand).

### 2.4 Cloning

Samples showing filarial co-infection (represented by mixed chromatograms) were cloned using the Zero Blunt™ TOPO™ PCR Cloning Kit (Thermo Fisher Scientific, USA) to extract separate sequences for both target organisms. Obtained plasmid DNA was purified from the bacterial culture using the GenElute™ Plasmid Miniprep Kit (Sigma-Aldrich, USA), and then sequenced using universal T7/SP6 primers.

### 2.5 Phylogenetic and statistical analysis

**Figure 1:**
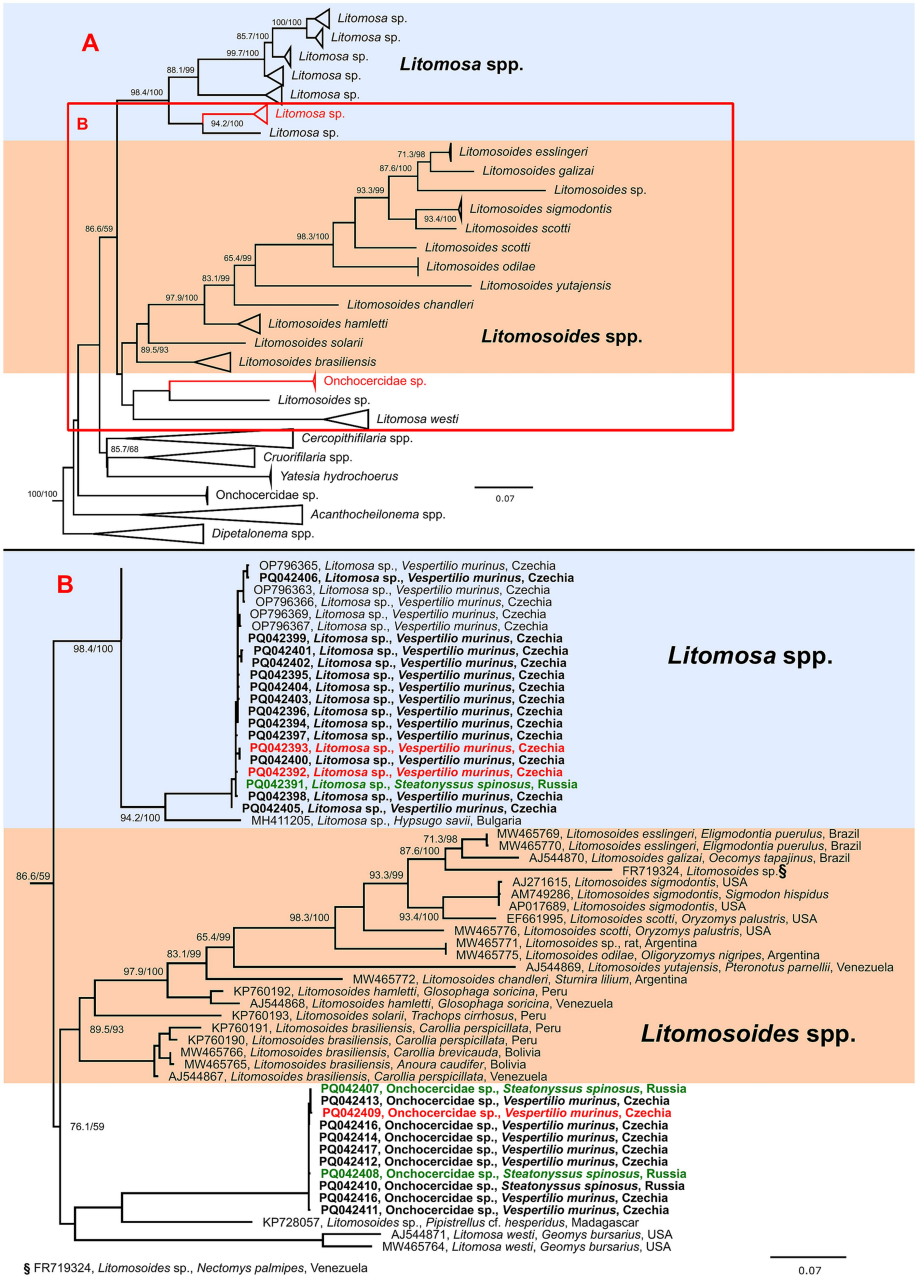
Schematic representation of a maximum likelihood phylogenetic tree based on the cytochrome oxidase c subunit I (*COI*) gene sequences of genera closely related to *Litomosa* and *Litomosoides* spp. **(A)** with detailed phylogeny of part of *Litomosa* spp., *Litomosoides* spp. and related clade containing Onchocercidae sp. **(B)**. The final length of the alignment was 638 bp and contained 182 sequences (27 originating from this study). The tree was constructed using the evolution model TIM3 + F + I + G4. Three sequences of *Breinlia robertsi* used as an outgroup are not shown. Sequences from this study are marked in bold and clones originating from the same sample are shown in matching colors (red or green). The scale bar indicates the number of nucleotide substitutions per site. Sequences are labeled with accession number, species, host and country of origin (where available). Bootstrap values (SH-aLRT/UFB) above the 80/95 threshold are also displayed.

Two phylograms of the *COI* gene were constructed. First, a phylogenetic tree covering the entire superfamily Filaroidea was built to confirm and specify the identity and phylogenetic position of the sequences from the present study. Based on this phylogeny, a detailed analysis of *Litomosa* spp., *Litomosoides* spp., and closely related genera was performed. For the initial analysis, all unique *COI* sequences longer than 300 bp available in the GenBank database were used, while representative sequences were used to construct the second phylogeny (for further details on the phylogenetic analysis, including number of sequences used, algorithm used, length of final alignments and evolution models chosen, see Figure 1; for a more detailed representation of the phylogenetic analysis with all sequences used, see Supplementary Figure S1) All phylogenies were inferred by IQ-TREE version 1.6.12 (17) and the best-fit evolution model selected based on the Bayesian information criterion, computed and implemented using ModelFinder (18). Branch supports were assessed by ultrafast bootstrap (UFBoot) approximation (19), and the Shimodaira-Hasegawa-like approximate likelihood ratio test (SH-aLRT) (20). Trees were then visualized and edited in FigTree v1.4.4^2^ and Inkscape 1.3.^3^

Prevalence of filarial infection was compared by testing the difference between two proportions, the Chi-square test being used to detect patterns of filarial infection distribution in all tissues excluding testes.

## 3 Results

### 3.1 Filariae in *Vespertilio murinus* bats: sequencing, genetic diversity, and phylogenetic analysis

Overall, 52.8% (66/125) of *V. murinus* tissue samples, 100% (5/5) of adult worms, and 44.4% (4/9) of ectoparasite-pooled samples proved positive for filariae using nested-PCR targeting partial *COI*. The PCR positive samples were successfully sequenced, with a total of 27 unique sequences obtained. According to BLAST analysis, 34 samples were identified as *Litomosa* sp., with the closest match being sequences of *Litomosa* sp. isolated from *V. murinus* in our previous study Pikula *et al.* (10; 98.7–100% identity, OP796365-71, Czech Republic), while 17 samples showed highest similarity to the *Eufilaria sylviae* sequence isolated from *Sylvia borin* (86.9–87.1% identity, MT800770, Lithuania), and were named as Onchocercid sp., i.e., an unspecified species of the family Onchocercidae. While sequence homology in *Litomosa* sp. ranged from 98.6 to 99.9%, the Onchocercid sp. sequences showed even higher similarity, ranging from 99.5 to 99.9%. The remaining 24 samples were identified as mixed infections with both the above-mentioned species based on analysis of mixed chromatograms. This was supported by cloning of two samples that showed mixed chromatograms (one from bat tissue, one from an ectoparasite) producing clean chromatogram sequences for both species.

All unique nucleotide sequences of the *COI* gene produced in this study were deposited in GenBank under accession numbers PQ042391-417, with numbers PQ042391, PQ042407 and PQ042408 for the Onchocercid sp. sequence, and numbers PQ042392, PQ042393, and PQ042409 for the *Litomosa* sp. being obtained from co-infected *S. spinosus* and *V. murinus* characterized by cloning and all others using nested-PCR.

Phylogenetic analysis of the *COI* of available Filaroidea sequences clearly showed that the sequences obtained in this study clustered with bat infecting genera of *Litomosa* and *Litomosoides* (data not shown). In the detailed phylogeny, the *Litomosa* sp. sequences formed a highly supported clade with *Litomosa* sp. from our previous study (10), while the sequences labeled as Onchocercidae sp. formed part of a cluster clade separate from *Litomosa* spp. and *Litomosoides* spp. composed of sequences from *Litomosa westi* and an unnamed *Litomosoides* sp. (Figure 1). As support for the branches for this species was not high, we could not place it in either *Litomosa* or *Litomosoides.*

### 3.2 Prevalence and distribution of filarial infection in *Vespertilio murinus*

Only four of the 27 vespertilionid bats tested (males *n* = 3, females *n* = 1; 14.8%) hosted adult filarial nematodes based on visual inspection during dissection. In total, we found 11 adult worms in the abdominal and thoracic (one case) cavities, ranging from one to four worms per bat. Five worms were tested (the rest being saved for future morphological analysis), and were genetically determined as *Litomosa* sp. (OP796365-71) with at least 99.6% identity. Two adult filarial worms were found within the uterine cavity of a mid-gestation pregnant female. Both uterine and fetal thoracic tissues tested positive by nested-PCR. None of the adult worms in this study were genetically identified as Onchocercid sp. All adult *Litomosa* sp. worms found in the body cavities of bats (*n* = 5) proved negative for presence of the bacterial symbiont *Wolbachia*.

Combined molecular screening for presence of microfilariae and adult nematodes revealed 85.2% of *V. murinus* as positive, with all bats with adult worms positive for the molecular presence of microfilariae. Prevalence of filarial larval infection was significantly higher than infection with adults (Difference test, *p* < 0.001). Molecular analysis of tissues revealed a prevalence of 81.5% (22/27) for *Litomosa* sp. and 51.9% (14/27) for Onchocercid sp., with Onchocercid sp. always present in bat bodies as a co-infection with *Litomosa* sp. (with one exception) at significantly lower prevalence (Difference test, *p* < 0.05).

The difference in microfilarial infection prevalence between males (80.0%) and females (91.7%) was not significant (Difference test, *p* = 0.395), even when examining individual filarial species (*Litomosa* sp. *p* = 0.551, Onchocercid sp. *p* = 0.130). Tissue analysis indicated parasites distributed throughout the body, i.e., presence of circulating microfilariae of both species, whether individually or as a mixed infection, was confirmed in all tissue types (see Table 2), usually affecting multiple organs of each individual and in all possible combinations. There was no significant difference in the distribution of filarial species in the various organs (χ^2^ = 10.625, *p* = 0.101). In addition, we tested the presence of microfilariae in the testicles of males and found a prevalence of 66.7%.

**Table 2:** Prevalence of filarial infection in organs of *Vespertilio murinus* bats (females *n* = 12, males *n* = 15).

	Sex	Testicles [%]	Heart [%]	Spleen [%]	Kidneys [%]	Liver [%]	Overall [%]
*Litomos*a sp.	Female	-	16.7	75.0	50.0	45.5	83.3
	Male	53.3	46.7	44.4	46.7	33.3	80.0
	Total	53.3	33.3	53.8	48.1	38.5	81.5
Onchocercid sp.	Female	-	25.0	25.0	33.3	9.0	41.6
	Male	60.0	46.7	11.1	26.7	20.0	60.0
	Total	60.0	37.0	15.4	29.6	15.4	51.9
Mixed infection	Female	-	0	0	8.3	0	33.3
	Male	46.7	40.0	11.1	20.0	20.0	60.0
	Total	46.7	22.2	7.7	14.8	11.5	48.1
Total prevalence		66.7	48.1	61.5	63.0	42.3	85.2

### 3.3 Identification of a potential vector mite

According to the nested-PCR analysis, microfilarial infection was limited to the ectoparasite *S. spinosus*, with four samples positive, the other pooled samples all proved negative. Three of the positive pooled samples harbored Onchocercid sp. DNA, and one showed mixed filarial infection. Molecular cloning of the latter sample confirmed simultaneous occurrence of both *Litomosa* sp. and Onchocercid sp. All positive pooled samples originated from Russia (Lukashino region, Western Siberia). Both negative pooled samples of *I. obscurus* were collected from the same bats as the two pooled samples of *S. spinosus* that proved positive for microfilariae.

## 4 Discussion

### 4.1 Filariae in *Vespertilio murinus* bats: sequencing, genetic diversity, and phylogenetic analysis

More than 20 species of *Litomosa* parasite have been described, seven of which have been recorded in European bats (though some have only been reported once), i.e., *L. aelleni* in Switzerland, *L. vaucheri* in Switzerland, *L. dogieli* in Europe, *L. filaria* in Europe, *L. beshkovi* in Bulgaria, *L. ottavianii* in Italy, and *L. seurati* in North Africa and southern France (21). However, these species have only been described morphologically, and molecular data on filarial nematodes of European bats remains scarce. Apart from the *Litomosa* sp. reported in our previous study (10), only one other *Litomosa* sequence has been reported from a European bat (MH411205; *Hypsugo savii*, Bulgaria). Our phylogenetic data revealed that the parasites recorded in our samples were closely related species and were unequivocally members of the genus *Litomosa*. The second species reported, here named Onchocercidae sp., formed part of a separate cluster, distinct from other *Litomosa* and *Litomosoides* spp., containing sequences of *Litomosa westi* (a parasite of Geomyid rodents in North America) (22) and an undescribed *Litomosoides* (KP728057, *Pipistrellus cf. hesperidus*, Madagascar). However, branch support was not high, and the cluster clearly changed position in different phylogenies (data not shown). Low bootstrap support, together with the absence of adult worms, did not allow us to firmly place the detected species within the filarial nematode taxonomy. Furthermore, the mentioned sequence of *Litomosoides* sp. (KP728057) was obtained from microfilariae, and no adult worms were ever found, meaning it could not be reliably assigned to *Litomosoides* spp. (5). Taken together, this suggests that there may be at least one other genus closely related to the genera *Litomosa* and *Litomosoides*. More molecular studies are needed to confirm possible new genera of bat-infecting filarial nematodes.

The distribution of *V. murinus* is quite extensive, ranging from Central Europe to Mongolia and Eastern Russia (23). Interestingly, while our necropsied bat samples originated from the Czech Republic and the *S. spinosus* mites positive for both detected parasites were collected in west Siberian Russia, we failed to detect any significant difference in relation to geographic origin of the sequences, with all samples clustering together in both parasites. This suggests that both parasites might be widespread and, consequently, their vector (or vectors) is also likely to be widespread (24). A similarly wide distribution range was also observed in *Dirofilaria repens* and *D. immitis*, filarial nematodes that affect dogs and other carnivores such as cats, wolves and foxes, where its distribution can be at least partially attributed to dog movements (25). Our study species, *V. murinus*, is a long-distance migrant capable of flying more than 1,500 kilometers southwest or southeast between its winter and summer roosts in regions with milder climates (26). Consequently, infectious agents can be spread via yet unknown vector between different locations over a wide geographic area. This species-specific aspect of the host bat species may also influence the parasite’s prevalence, with differing abilities of bat species to move between habitats resulting in greater or fewer encounters with each other and with blood-sucking vectors that may only be present in certain regions or habitats (27).

### 4.2 Prevalence and distribution of filarial infection in *Vespertilio murinus*

In filarioid nematodes, larvae released from females enter the host’s lymphatic system and blood vessels as microfilariae. At this point, they are ready to be ingested by an ectoparasitic vector, in which they develop into infective L3 larvae that can then infect a new host as it feeds on another bat. In the new host, they continue development into L4 larvae, migrating through the bat’s body to their definitive site of maturation and dwelling (28, 29). As the molecular detection method used in the present study is not able to distinguish different larval stages, tested organs and mites could theoretically be positive due to different filarial developmental stages. There also appears to be no single target organ providing higher probability of microfilarial detection in *V. murinus*. Instead, the overall prevalences documented (i.e., ~82% *Litomosa* sp., ~52% Onchocercid sp., ~48% mixed infection) suggest the common occurrence of these parasites throughout *V. murinus*. Discrepancies in infection prevalence based on presence of adult worms and/or molecular larval detection may result from the difficulty of finding the minute thread-like filarial nematodes during dissection, differences in infection stages between individual bats, and differences in survival of microfilarial and adult nematodes within the host body (30).

Findings of 11 *Litomosa* sp. worms in the present study agree with the previous knowledge that adult filarial worms are typical cavity dwelling nematodes of small mammals, including bats (21, 31–37). However, they may also be parasites of subcutaneous tissues (28).

Unfortunately, we were not able to find adult Onchocercid sp. worms in this study, and the site where to look for these parasites remains elusive. Nevertheless, careful techniques of microdissection and microscopic tissue squash and wet mount examination should be used during bat necropsies in the future. Interestingly, two adult *Litomosa* sp. worms were discovered inside the uterus of a pregnant *V. murinus* female, and fetal tissues were also positive for filarial DNA in this case, meaning that the *Litomosa* sp. microfilariae can pass through the uterine wall and placenta (38–40). High ectoparasite loads and abundant bat aggregations typical for bat nursery colonies (41) may further increase opportunities for vector-borne cycling based on bat offspring infected transplacentally, similar to canine puppies (42), possibly contributing to the observed high filariasis prevalence.

Based on our previous finding of microfilariae in the bat’s semen (10) suggesting polygynous mating of *V. murinus* as a possible route of microfilarial transmission of the *Litomosa* sp. nematode, we expected host-sex differences in the infection prevalence (43). Likewise, some other aspects such as the social behavior of host males segregating from females for most of the year and their territorial individual roosting could influence the risk of filarial infection (44). However, this prediction of host-sex-related differences was not confirmed. As shown in Table 2, testicular tissues were rather commonly positive (~67%) for single and mixed microfilarial infections by both parasite species detected in the present study. It remains unclear whether this transmission of microfilariae occurs and to what an extent, and whether it decreases semen quality and challenges the success of reproductive events in females after mating (10). An alternative route of pathogen transmission could be advantageous, for example, during the period of limited exposure to arthropods (45–47) which are an essential part of the life cycle of filarial nematodes (30).

Since filarial nematodes can contain *Wolbachia* endosymbionts, we investigated their presence in adult worms using PCR screening. Interestingly, *Wolbachia* was not detected in *Litomosa* sp., despite claims that it is essential for the biology of its filarial host (e.g., see Casiraghi *et al.*) (11) and it having been confirmed in other species of the genus, e.g., *L. westi* from rodents (12) and most species of *Litomosoides* parasitising bats or rodents (36). However, data obtained both in this study and in Madagascar (21) show that *L. westi* is phylogenetically different from other known *Litomosa* parasites; a feature also supported by the lack of *Wolbachia* endosymbionts in our bat-infecting parasite. Support for the loss of *Wolbachia* during filarial evolution is growing (36); for example, our own findings of absence are consistent with results for *L. chiropterorum* (36) and the single species *Litomosoides yutajensis* (12). A possible explanation may be secondary loss during evolutionary development in some filarial nematode species (1). In any case, distribution of *Wolbachia* within the Onchocercidae appears to be inconsistent, and even among closely related filarial nematodes, the picture remains complicated.

### 4.3 Identification of a potential vector mite

Very little is known about the filarial nematode life-cycle transmission phase in bats, and their invertebrate vectors are poorly understood (5). Bats host a wide variety of ectoparasites (45, 48), including those that we tested using molecular methods, i.e., fleas (Siphonaptera: Ischnopsyllidae), ticks (Ixodida: Argasidae: *Carios*), and mites of the genus *Steatonyssus* (Mesostigmata: Macronyssidae). In this study, we showed that only *S. spinosus* mites were positive for presence of microfilarial DNA. As fleas collected from the same individual tested negative, this suggests that *S. spinosus* may be a potential vector. This may be supported by previous suggestion that mites of the order Mesostigmata may be potential vectors of larval filariae stages (36), while macronyssid mites are thought to be a vector of *Litomosoides* in rodents, marsupials and bats (33, 49, 50). This hypothesis of transmission by macronyssid mites is also supported by experimental introduction of *Ornithonyssus bacoti* onto the microfilaraemic Parnell’s mustached bat (*Pteronotus parnellii*) (34) and the Jamaican fruit bat (*Artibeus jamaicensis*) (30). The most common ectoparasite of *V. murinus*, *S. spinosus*, is recorded throughout most of the species’ range (16, 51, 52) and is characterized by a high degree of adherence to the host and relatively strong host specificity (51). They parasitise their hosts in the summer roosts (47), with some species becoming permanent parasites (53). Moreover, a related *Steatonyssus* species, *S. periblepharus*, has recently been suggested as a novel potential vector of the bat parasite *Trypanosoma dionisii* (54). Interestingly, such wing membrane mites may also serve as vectors of some other infectious agents, such as the white-nose syndrome fungus (55). Nevertheless, the sole presence of DNA in *S. spinosus* does not prove that this ectoparasite serves as a vector of the detected parasites, and experimental studies are needed to assess its role in the epidemiology of bat infecting filarial nematodes.

## 5 Conclusion

We detected highly prevalent single and mixed infections with two filarial species in *V. murinus*. The first parasite, identified as *Litomosa* sp., has already been reported in our previous study (10), while the second could only be characterized as a species of the Onchocercidae family using molecular methods as adult worms were not discovered during necropsies of bat cadavers. Phylogenetic analysis of parasite *COI* sequences originating from bats sampled in the Czech Republic, and from *S. spinosus* mites collected on *V. murinus* in Russia, suggests extensive spatial distribution of both filarial species. As *S. spinosus* mites tested positive for microfilarial DNA of both parasitic worms, these mites may serve as vectors for these filarial infections. Our data strongly suggest that a taxonomic revision of bat-infecting filarial nematodes is needed.

## Data Availability

The datasets presented in this study can be found in the GenBank database (https://www.ncbi.nlm.nih.gov/genbank/) under accession numbers PQ042391-417.

## Footnotes

^1^ https://www.ncbi.nlm.nih.gov/genbank/

^2^ http://tree.bio.ed.ac.uk/software/figtree/

^3^ https://inkscape.org/
