# Supplementary material for: Filariasis of parti-colored bats: phylogenetic analysis, infection prevalence, and possible vector mite identification
Source: Front Vet Sci. 2025 Mar 5;12:1546353. doi: 10.3389/fvets.2025.1546353 (PMC11920757; doi:10.3389/fvets.2025.1546353)
Supplement: Supplementary file 1 [file Data_Sheet_1.pdf]

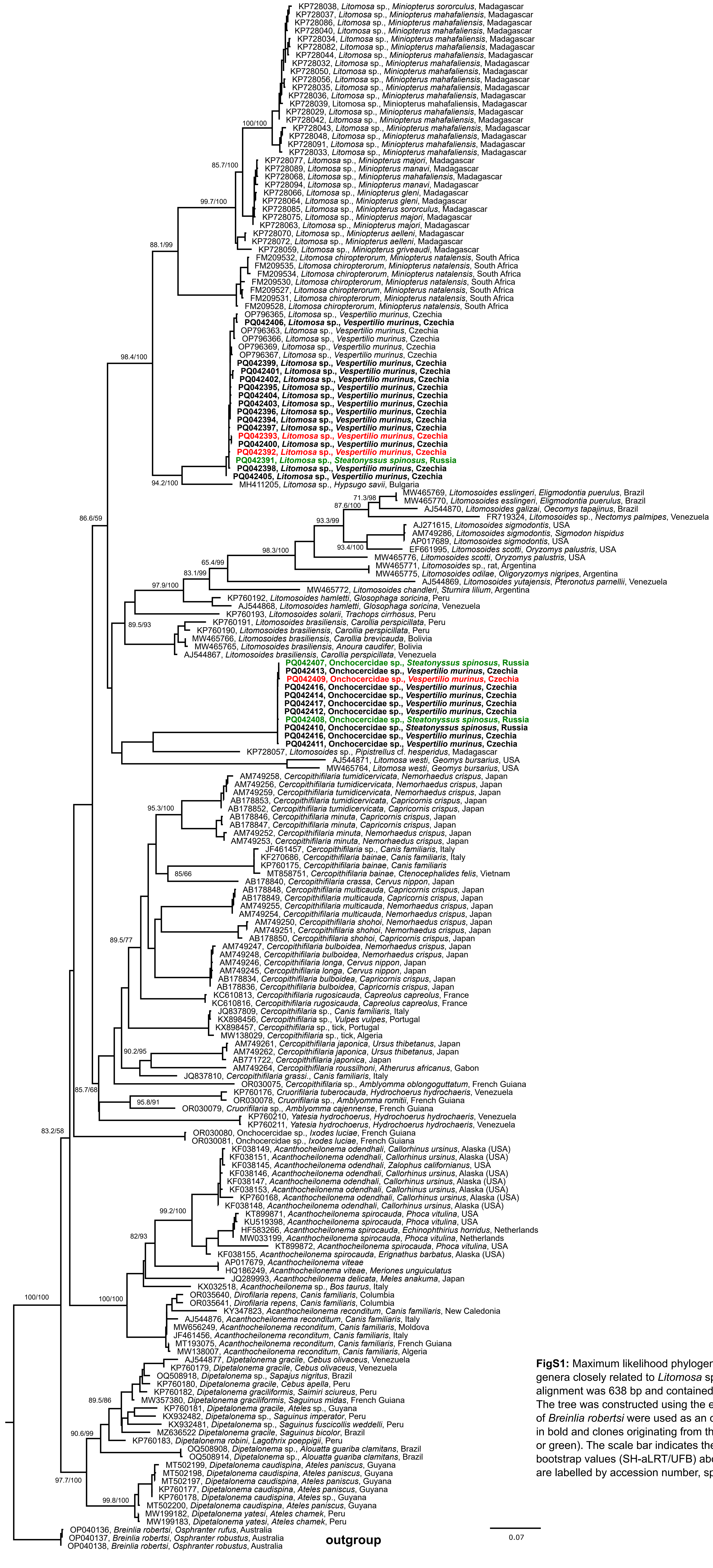

FigS1: Maximum likelihood phylogenetic tree based on the COI gene sequences of genera closely related to *Litomosa* spp. and *Litomosoides* spp. The final length of the alignment was 638 bp and contained 182 sequences (27 originated from this study). The tree was constructed using the evolution model TIM3+F+I+G4. Three sequences of *Breinlia robertsi* were used as an outgroup. Sequences from this study are marked in bold and clones originating from the same sample are shown in matching color (red or green). The scale bar indicates the number of nucleotide substitutions per site. The bootstrap values (SH-aLRT/UFB) above the 80/95 threshold are displayed. Sequences are labelled by accession number, species, host, and country of origin (if available).
